# Protective effects of gallocatechin gallate against ultraviolet B induced skin damages in hairless mice

**DOI:** 10.1038/s41598-022-05305-9

**Published:** 2022-01-25

**Authors:** Yue-Yue Sheng, Jing Xiang, Jian-Liang Lu, Jian-Hui Ye, Zi-Jiu Chen, Jian-Wen Zhao, Yue-Rong Liang, Xin-Qiang Zheng

**Affiliations:** 1 Tea Research Institute, Zhejiang University, Hangzhou, 310058 China; 2 Qiandaohu Beauti-Health Medical Institute Ltd, Hangzhou, 311701 China

**Keywords:** Biochemistry, Drug discovery, Climate sciences, Natural hazards, Health care, Medical research, Signs and symptoms

## Abstract

Epigallocatechin gallate (EGCG) has the effect to protect skin from ultraviolet B (UVB) induced damages, but it is unstable under ambient conditions, being susceptible to become brown in color. Gallocatechin gallate (GCG), an epimer counterpart of EGCG, is more stable chemically than EGCG. The potential effects of GCG against UVB-induced skin damages has not been available. The objective of this study was to investigate the protective effects of GCG against UVB-induced skin photodamages. GCG was topically applied on the skin of hairless mice at three dosage levels (LL, 12.5 mg/mL; ML 25 mg/mL; HL, 50 mg/mL), with EGCG and a commercially available baby sunscreen lotion SPF50 PA^+++^ as control. The mice were then irradiated by UVB (fluence rate 1.7 µmol/m^2^ s) for 45 min. The treatments were carried out once a day for 6 consecutive days. Skin measurements and histological studies were performed at the end of experiment. The results show that GCG treatments at ML and HL levels inhibited the increase in levels of skin oil and pigmentation induced by UVB irradiation, and improved the skin elasticity and collagen fibers. GCG at ML and HL levels inhibited the formation of melanosomes and aberrations in mitochondria of UVB-irradiated skin in hairless mice. It is concluded that GCG protected skin from UVB-induced photodamages by improving skin elasticity and collagen fibers, and inhibiting aberrations in mitochondria and formation of melanosomes.

## Introduction

Skin is the body's first line of defense and it acts as a shield to protect the body from environmental stress. It consists of epidermis, dermis and the subcutaneous tissue. Ultraviolet B (UVB) is the light with a wavelength 280–320 nm. Although the UVB irradiation is less than 5% of total sunlight radiation, it is very harmful to skin due to high absorption by biomolecules such as DNA and proteins. UVB elicits alterations in the epidermal level of the skin^1^ and it causes damages to the skin by generating reactive oxygen species (ROS) which induce mitochondrial aberrations, DNA damage and apoptosis in UVB irradiated skins^2,3^. Prolonged exposure of the skin to UVB irradiation induced degradation of elastic fibers, resulting in skin laxity, dryness, deep wrinkling and pigmentation^4,5^. Skin melanocytes can synthesize melanin to protect keratinocytes from UVB irradiation^6,7^. It is interesting to search for natural sunscreen agents to protect skin from photodamages induced by UVB irradiation.

Catechins are a group of bioactive compounds found in tea leaf and they have attracted much attention due to their physiological activities such as anti-inflammatory, antioxidant and anti-cancer^8–11^. Epigallocatechin gallate (EGCG) is the most abundant catechins, accounting for more than 50% of the total catechins in fresh tea leaves and unfermented green tea^12^. Gallocatechin gallate (GCG) is the spatial isomer of EGCG^13^. Both EGCG and GCG have strong radical-scavenging activity. In the fresh leaves of cultivated tea plants, there is trace amount of GCG. However, GCG is abundant in dry tea because of epimerization of EGCG induced by heating during tea processing^13,14^. Recent study revealed that GCG is abundant in some wild tea plants, such as *Camellia ptilophylla* and *Camellia assamica* var. *Kucha*^15–18^.

EGCG was proved to have protective effects against UVB-induced skin damages^19^, but it is susceptible to illumination, oxygen and high temperature, resulting in browning in color, which is undesirable in cosmetic products^20^. GCG, an epimer counterpart of EGCG, is less susceptible to oxygen, heating and illumination^20^. GCG showed stronger antioxidant activity than EGCG in certain circumstance^21^. It was reported that GCG has stronger free radical scavenging activity than EGCG at low concentration^22–24^. However, the protective effect of GCG against UVB-induced skin damages has not been available. This study aims to explore the anti-UVB effect of GCG on skin of hairless mice, so as to provide a useful information for developing sunscreen agents against UVB-induced photodamages or photoaging using GCG as ingredient.

## Materials and methods

### Reagents and equipment

Medical vaseline was purchased from Zhengmao Petrochemical Co. Ltd (Maoming City, China); EGCG (97% purity) and GCG (98% purity) (Fig. S1) were purchased from Ningbo Simingshan Biological Technology Co. Ltd (Yuyao City, China) and they were mixed with the medical vaseline at designed experimental concentration; Water Baby sunscreen lotion with SPF50 PA^+++^ (Bayer HealthCare LLC, Whippany, NJ, USA) was used as a positive control.

The major equipment used in the test included Ultraviolet Radiometer (Optical Instrument Factory, Beijing, China), transmission electron microscope (TEM) (Hitachi Model H-7650, Hitachi LTD, Tokyo, Japan); CBS-1800 Skin Analyzer (Wuhan Bose Electronic Co., Ltd, Wuhan, China); AB265-S Electronic Balance [Mettler Toledo (China), Shanghai, China]. The UVB was supplied by UVB lamps (Model BLE-1T158, Spectronics corp., Westbury, NY, USA) which emits UV in the range of 254–320 nm, with a peak at 297 nm.

### Experimental animals

Five-week-old BALB/c hairless mice used in this study were purchased from Shanghai Slack Laboratory Animal Co., Ltd (Shanghai, China, No. SCXK (Hu) 2017-0005). The mice were housed under a 12 h light /12 h dark cycle at room temperature. During the experimental period, the mice were free to eat feed and drink water. All the animal experiment procedures were approved by the Laboratory Animal Welfare and Ethics Committee of Zhejiang University (Ethics code ZJU20170077). The experiments were performed in accordance with the criteria of the “Guide for the Care and Use of Laboratory Animals” (NIH publication 86-23, revised 1985) and the ARRIVE guidelines (https://arriveguidelines.org).

The purchased hairless mice were acclimated under the above controlled conditions for two weeks and then divided into blank control group (BC, without vaseline, GCG and UVB), negative control group (NC, topically treated with vaseline and then irradiated by UVB), positive control group (PC, topically treated with water baby sunscreen SPF50 PA^+++^ and then irradiated by UVB), EGCG group [EGCG, treated with EGCG (25 mg/mL) and then irradiated by UVB], low level GCG group [LL, topically treated with GCG (12.5 mg/mL) and then irradiated by UVB], medium level GCG group [ML, topically treated with GCG (25 mg/mL) and then irradiated by UVB], high level GCG group [HL, topically treated with GCG (50 mg/mL) and then irradiated by UVB]. Each group contained 13 male and 13 female hairless mice.

### Drug application and UVB irradiation

GCG and EGCG were mixed with vaseline according to the above designed concentrations. The drugs including NC, PC, EGCG and GCG at designed concentration were topically smeared on the skin of the tested hairless mice at a dosage 20 µL/cm^2^. At 30 min after the drug application, the hairless mice, except BC group, were irradiated with UVB (fluence rate 1.7 µmol/m^2^ s) for 45 min. The drug application and UVB irradiation were performed once a day for 6 consecutive days. On the seventh day, skin quality indicators including contents of oil, moisture, pigmentation and collagen fiber, and also skin elasticity were determined using CBS-1800 Skin Analyzer according to its instructions. The skin analyzer collects images of the skin to be detected using a CCD camera equipped with polarizing light and transfers the images through a progressive scan image sensor to the data processor where the levels of oil, moisture, pigmentation, collagen fibers and elasticity are evaluated by comparing them with built-in references in the data bank. The skin analyzer gave the indicators with a measurement unit %, which were used to assess the skin quality based on the values obtained from health skins. Then the tested hairless mice were sacrificed and the skins (1 cm^2^) were sampled and fixed for 4 h in 2.5% glutaraldehyde dissolved in phosphate buffer (0.1 mol/L, pH 7.0) for transmission electron microscopy (TEM) observation.

### Transmission electron microscopy (TEM)

The above fixed skin samples were washed in the phosphate buffer (0.1 mol/L, pH 7.0) for 15 min, and the operation was repeated for three times. The washed samples were fixed in 1% OsO_4_ dissolved in phosphate buffer (0.1 mol/L, pH 7.0) for 1.5 h and then washed three times in the phosphate buffer as before. The fixed samples were successively dehydrated in a graded series of ethanol (30%, 50%, 70%, 80%, 90% and 95%) for 15 min at each step, and finally dehydrated by 100% ethanol for 20 min. The dehydrated samples were immersed in pure acetone for 20 min, suspended in a mixture of acetone and Spurr resin (1:1) for 1 h at room temperature, then in mixture of acetone: Spurr resin (1:3) for 3 h, and finally in pure Spurr resin overnight.

The above specimens were placed in 1 mL eppendorf tubes containing Spurr resin and heated at 70 °C for more than 9 h. The specimen was sectioned using LEICA EM UC7 ultramicrotome and the sections were stained by uranyl acetate and alkaline lead citrate for 10 min respectively, and finally observed and photographed on Hitachi Model H-7650 TEM.

### Assessment of melanosomes

The melanin content of the specimens was evaluated by counting the number of melanosomes in 10 μm × 10 μm TEM photographs with a constant magnification (× 4000). Five sections per treatment, with three TEM photographs each section, were counted and evaluated.

### Assessment of mitochondrial damage index

**Figure 1:**
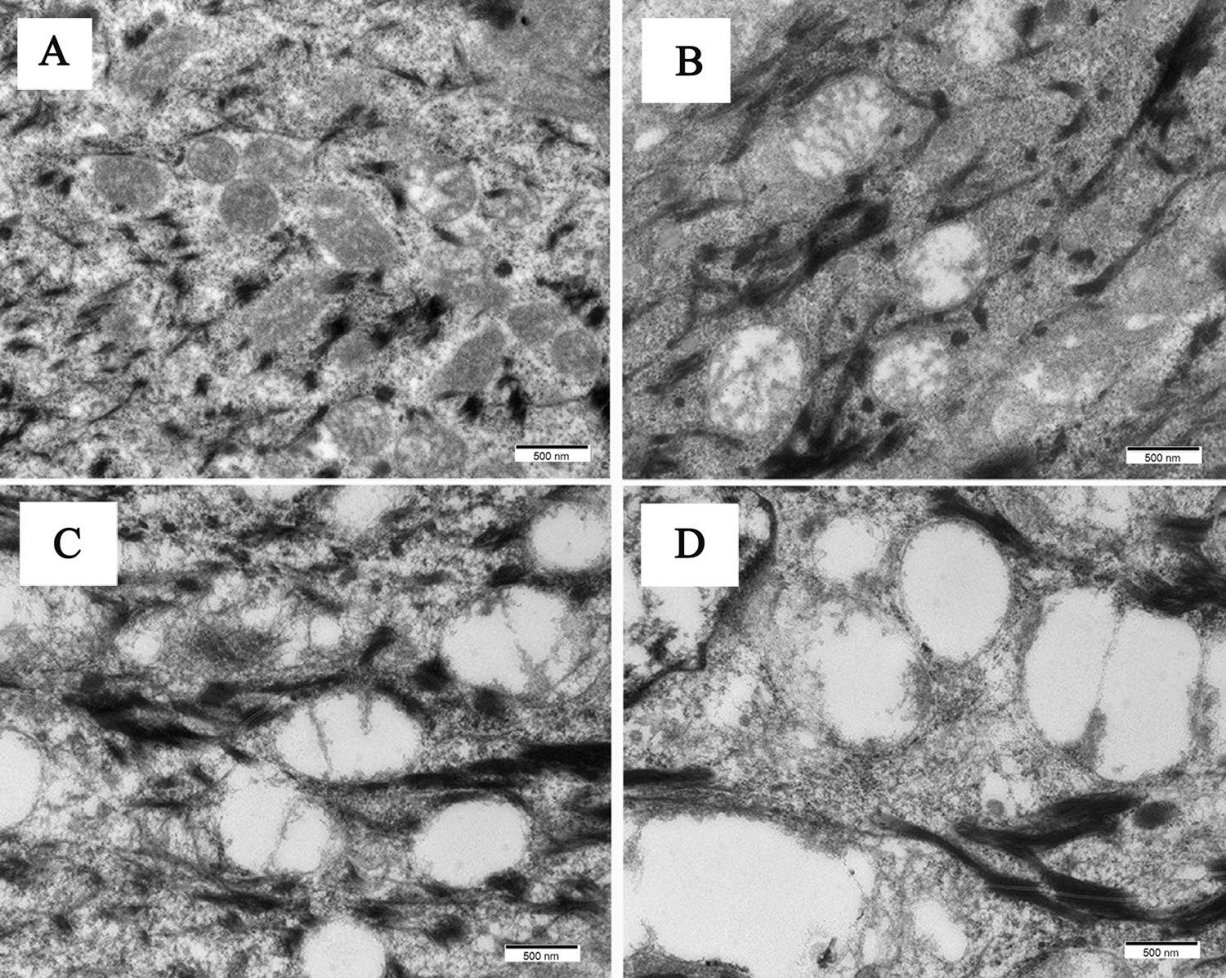
Semiquantitative scale of mitochondria injury degree. (**A**) scale 0, no damage; (**B**) scale 1, minimal damage; (**C**) scale 2, moderate damage; (**D**) scale 3, heavy damage.

**Figure 2:**
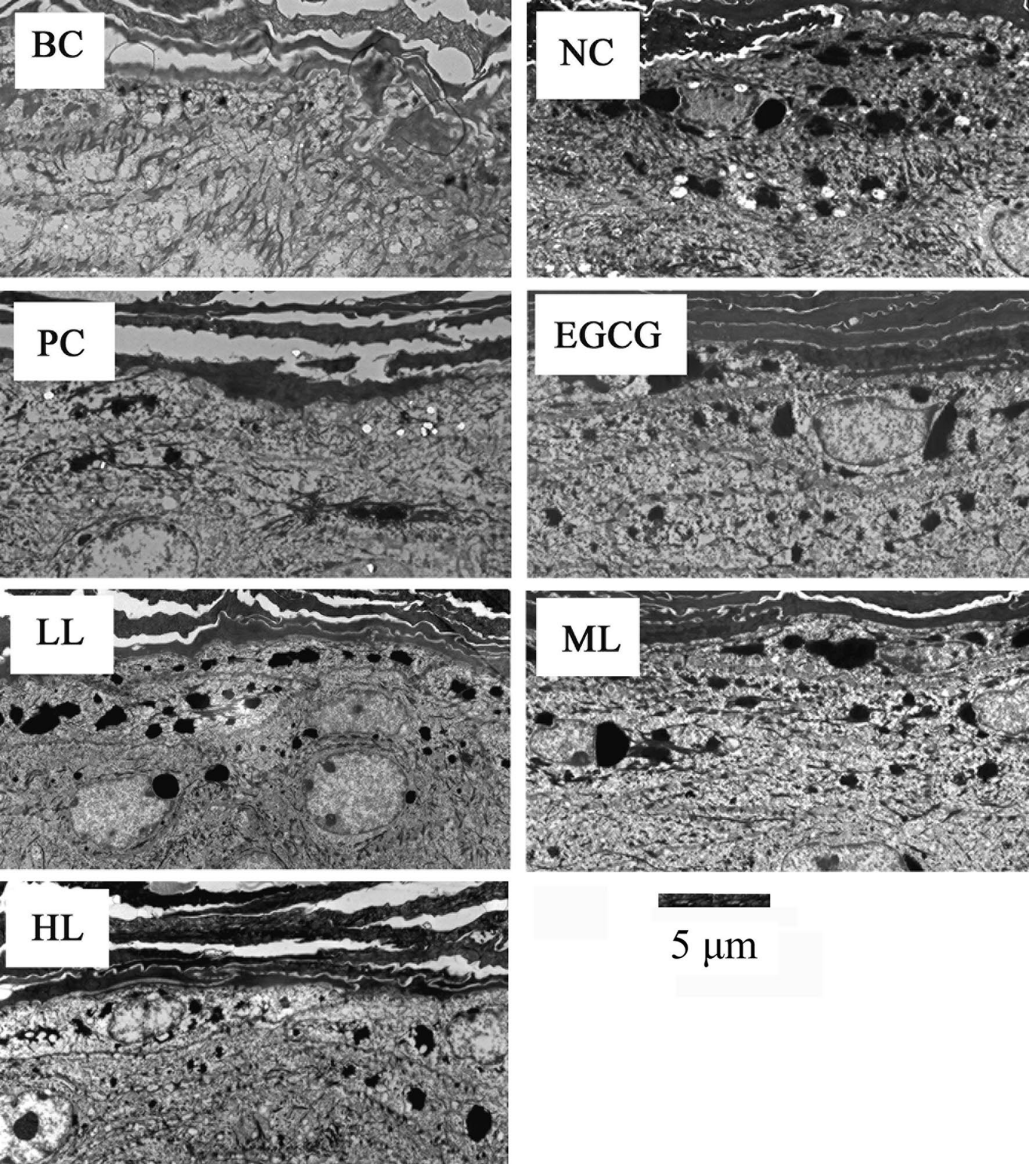
Effects of GCG on UVB-induced melanosome formation in female hairless mice skin. *BC* blank control, *NC* negative control, *PC* positive control, *EGCG* EGCG treatment, *LL* low level GCG treatment, *ML* medium level GCG treatment, *HL* high level GCG treatment, dark dots: melanosomes.

Mitochondria damage index was evaluated by semiquantitative scale under constant magnification (× 30,000) of TEM. The following scale was used: 0, no damage in which the mitochondria were rich in mitochondrial matrix and cristae as in Fig. 1A; 1, minimal damage in which the mitochondria were swollen and the cristae were decreased as in Fig. 1B; 2, moderate damage in which the swollen mitochondria had a few cristae as in Fig. 1C; and 3, heavy damage in which the swollen mitochondria had no cristae. Five sections each treatment were reviewed by three researchers and the mean value was expressed as mitochondrial damage index.

### Data analysis

The data were analyzed by software SPSS Statistics V20.0. Mean values of the quantitative data were expressed and the differences between various treatments were tested by T-test at p = 0.05.

## Results

### Effect of GCG on skin damage induced by UVB

Testing results of skin quality indicators by skin analyzer show that compared to the blank control (BC), UVB irradiation (NC, negative control) induced an increase in levels of skin oil, moisture and pigmentation, accompanying with a decrease in elasticity and collagen fibers in hairless mice skin. Compared to NC and positive control (PC, sunscreen lotion), indicators of skin oil, pigmentation, elasticity and collagen fibers were significantly improved in treatments of EGCG, medium level (ML) and high level (HL) of GCG (Table 1), suggesting that both GCG and EGCG have protective effects against UVB-induced photoaging in hairless mice skin.

**Table 1:** Effect of GCG and EGCG on skin quality indicators after UVB irradiation (%).

Treatment	Oil	Moisture	Pigmentation	Elasticity	Collagen fibers
BC	4.80 ± 2.49	12.50 ± 3.32	37.00 ± 7.70	38.80 ± 8.93	75.40 ± 2.88
NC	15.68 ± 5.86^§^	19.17 ± 6.56^§^	39.40 ± 7.02	36.63 ± 6.45	52.50 ± 8.69^§^
PC	16.75 ± 3.15^§^	30.14 ± 5.87^§^*	51.50 ± 6.72^§^*	37.63 ± 6.19	41.86 ± 6.64^§^*
EGCG	5.00 ± 2.28*	32.60 ± 11.91^§^*	46.00 ± 9.03^§^*	63.80 ± 7.01^§^*	77.83 ± 8.30*
LL	16.00 ± 3.22^§^	19.40 ± 5.68^§^	40.80 ± 5.54^§^	35.00 ± 7.14	53.40 ± 8.82^§^
ML	10.60 ± 4.16^§^*	15.40 ± 5.03*	30.33 ± 4.63^§^*	56.60 ± 15.21^§^*	72.17 ± 10.34*
HL	10.00 ± 4.05^§^*	12.00 ± 6.86*	33.40 ± 1.67^§^*	41.40 ± 3.21*	72.67 ± 3.93*

*BC* blank control, *NC* negative control, *PC* positive control, *EGCG* EGCG treatment; *LL* low level GCG treatment, *ML* medium level GCG treatment, *HL* high level GCG treatment.

^§^Being significantly different from BC at p = 0.05.

*Being significantly different from NC at p = 0.05.

### Effect of GCG on melanosome formation induced by UVB

**Figure 3:**
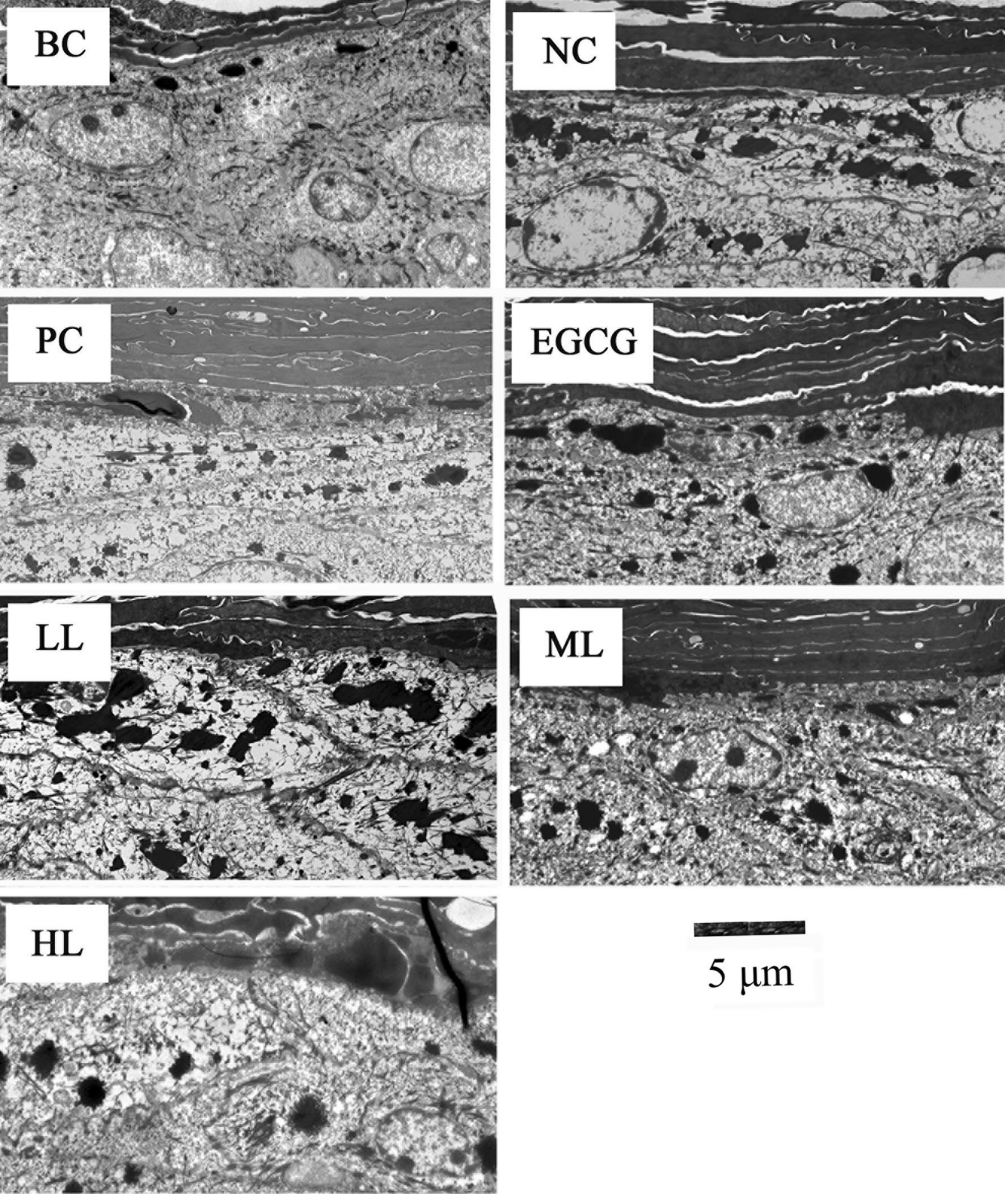
Effects of GCG on UVB-induced melanosome formation in male hairless mice skin. *BC* blank control, *NC* negative control, *PC* positive control, *EGCG* EGCG treatment, *LL* low level GCG treatment, *ML* medium level GCG treatment, *HL* high level GCG treatment, dark dots: melanosomes.

TEM observation and quantitative study show that a few melanosomes were observed in BC both female and male mice and the melanosomes were significantly increased by UVB irradiation in NC (Figs. 2 and 3, Table 2). However, the melanosomes were significantly less in PC and the treatments of EGCG and GCG (LL, ML, HL) in both female and male mice skins, compared to NC (Figs. 2 and 3, Table 2), which were consistent with the pigmentation results in Table 1. These indicate that UVB irradiation stimulated melanosome formation in hairless mice skin, and sunscreen lotion, EGCG and GCG had suppressive effects on UVB-induced melanosome formation.

**Table 2:** Effect of various treatments on UVB-induced formation of melanosomes.

Treatment	BC	NC	PC	EGCG	LL	ML	HL
**Melanosomes**							
Male	2.73*	15.33^§^	6.27^§^*	8.53^§^*	14.93^§^	8.60^§^*	7.27^§^*
Female	3.53*	15.13^§^	6.27^§^*	7.80^§^*	15.07^§^	8.47^§^*	7.87^§^*

^§^Being significantly different from BC at p < 0.05.

*Being significantly different from NC at p < 0.05.

### Effects of GCG on UVB-induced mitochondrial damage

**Figure 4:**
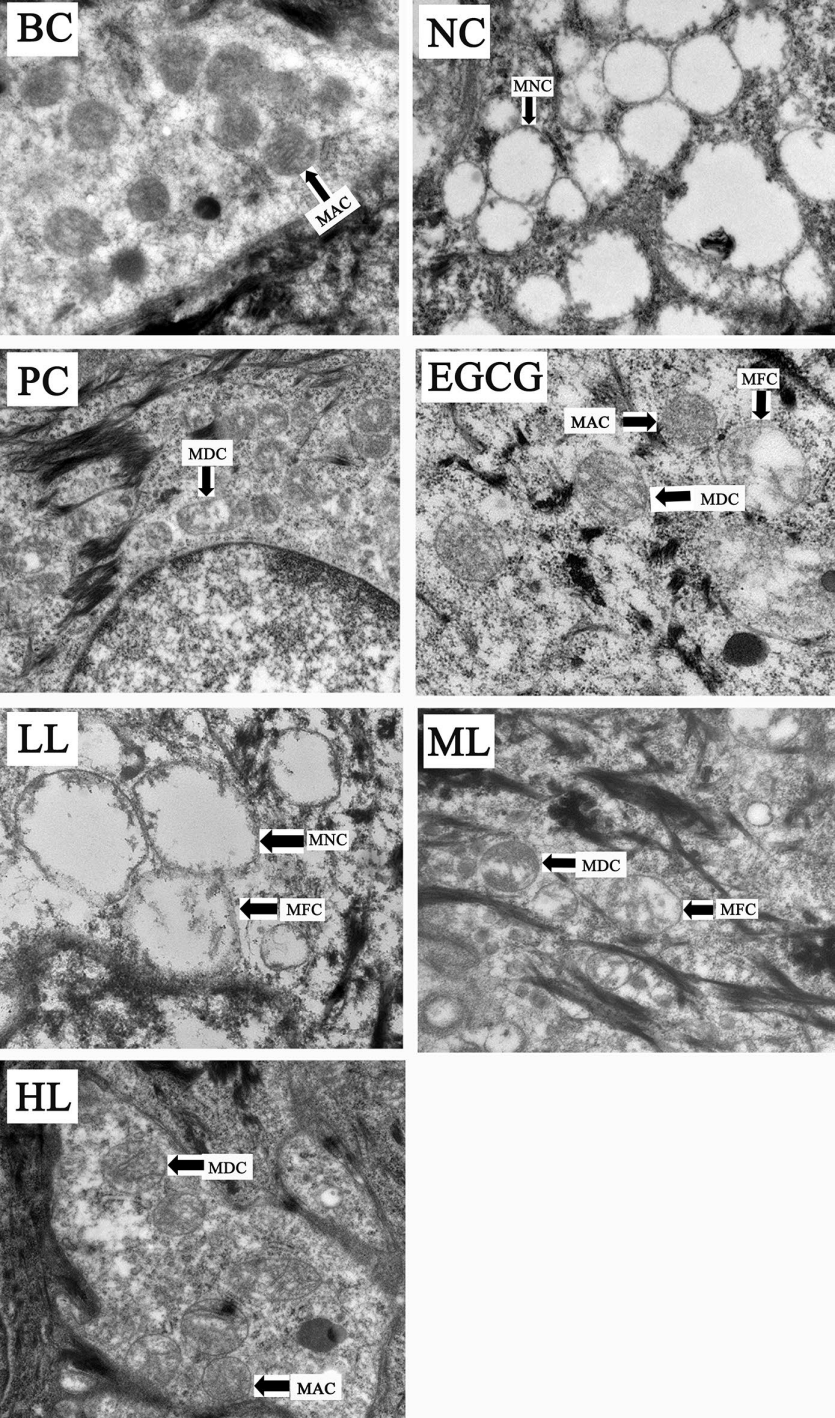
Effect of GCG on UVB-induced damage of mitochondria in skin of female hairless mice. *BC* blank control, *NC* negative control, *PC* positive control, *EGCG* EGCG treatment, *LL* low level GCG, *ML* medium level GCG, *HL* high level GCG, *MAC* mitochondrion with abundant cristae, *MDC* mitochondrion with decreased cristae, *MFC* mitochondrion with few cristae, *MNC* mitochondrion without cristae.

**Figure 5:**
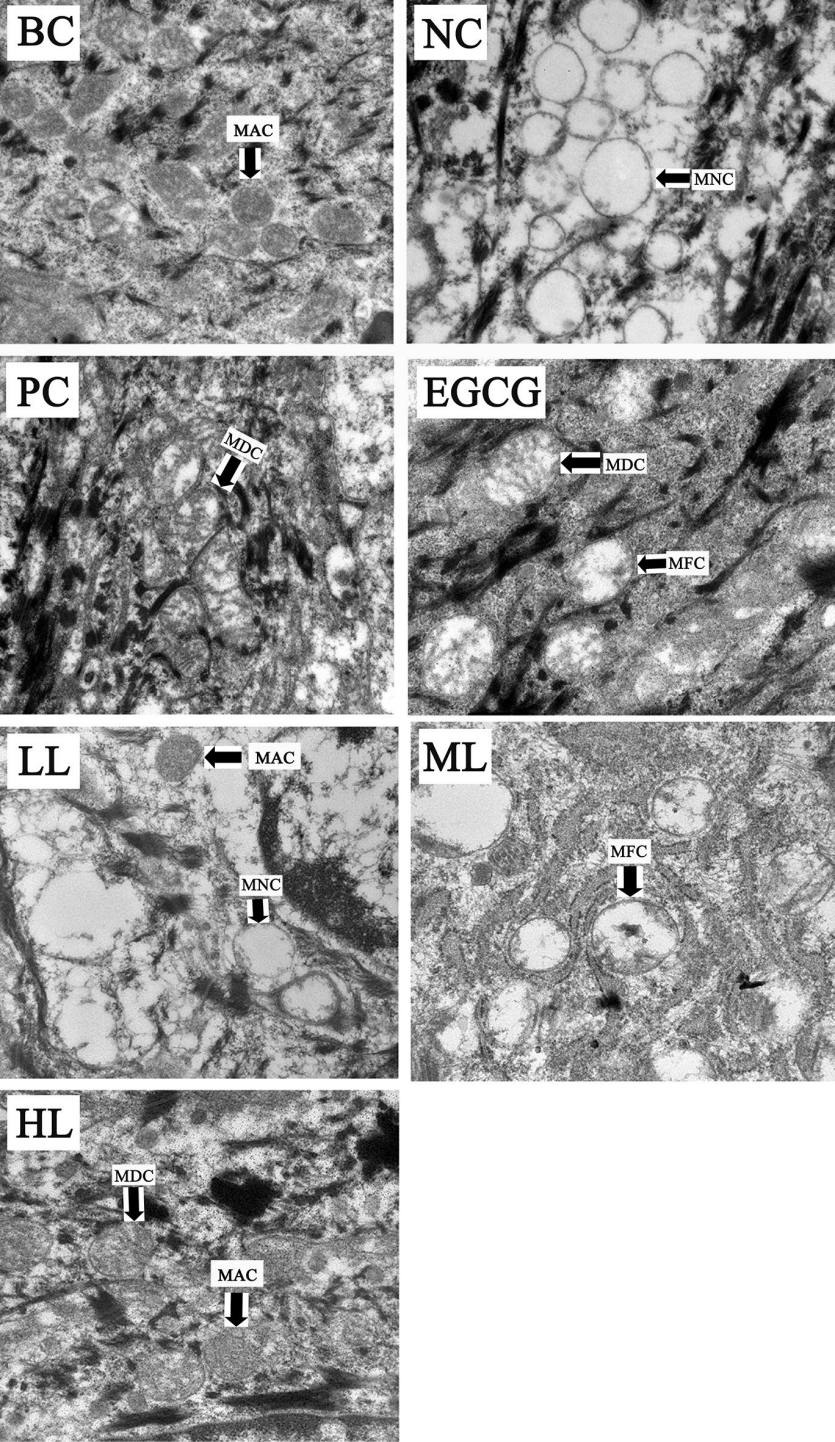
Effect of GCG on UVB-induced damage of mitochondria in skin of male hairless mice. *BC* blank control, *NC* negative control, *PC* positive control, *EGCG* EGCG treatment, *LL* low level GCG, *ML* medium level GCG, *HL* high level GCG, *MAC* mitochondrion with abundant cristae, *MDC* mitochondrion with decreased cristae, *MFC* mitochondrion with few cristae, *MNC* mitochondrion without cristae.

The UVB-irradiation induced changes in mitochondrial structure of skin cells in both female and male mice (Figs. 4 and 5, BC, NC). Before UVB-irradiation, the mitochondria were rich in mitochondrial matrix and cristae (Figs. 4 and 5, BC). The mitochondrial matrix and cristae were markedly reduced in mitochondria of NC. Like PC and EGCG, mitochondria in treatments of ML and HL of GCG contained more mitochondrial matrix and cristae than NC. However, LL had little effects on the reduction of mitochondrial matrix and cristae induced by UVB-irradiation (Figs. 4 and 5, Table 3). These suggest that GCG showed protective effects against mitochondria damages induced by UVB-irradiation in dose dependent manner.

**Table 3:** Mitochondria damage index of various treatments.

Treatment	BC	NC	PC	EGCG	LL	ML	HL
**Mitochondria injury index**							
Male	0.07*	2.60^§^	1.33^§^*	1.53^§^*	2.33^§^	1.67^§^*	1.40^§^*
Female	0.20*	2.67^§^	1.40^§^*	1.60^§^*	2.33^§^	1.73^§^*	1.40^§^*

*Being significantly different from BC at p < 0.05.

^§^Being significantly different from NC at p < 0.05.

## Discussion

Skin photoaging is a complex process involving light stress like UVB. Healthy skin is biomechanically resilient, highly elastic, and characterized histologically by strong interdigitation of rete ridges, abundant organized fibrillar collagen, and plentiful arrays of elastic fibers^25^. Skin elasticity depends on the condition of the extracellular matrix (ECM), which consists of primarily collagen. Collagen is an abundantly available protein structure in the ECM which is found in connective tissues of the human organs such as skin and is responsible for three-dimensional microstructure sustainability^26^. There is a total of 28 different collagen types, in which type I (Col-I) is the most commonly found in the body^27^. The Col-I monomers form triple-helical collagen structures through intertwining to form collagen fibrils, which are then assembled in bundles known as collagen fibers^28^. Collagen provides mechanical support in many tissues of mammals and provides tensile strength in the skin through its widespread molecular line formation^29^. Drugs with potential to improve collagen will be beneficial to skin health. Extract of *Foeniculum vulgare* (FV) significantly increased the production of collagen and elastin, showing dose-dependent decrease in production of reactive oxygen species (ROS) but increase in the expression of cytoprotective antioxidants such as glutathione, and significantly quenching UVB-induced phosphorylation of extracellular regulated protein kinases (ERK) and p38 mitogen-activated protein kinase (MAPK) in normal human skin fibroblasts ^30^. Polyphenols in combination with short exposure to UVA increased extracellular matrix deposition of elastin and collagen and improved skin properties^31,32^. Present study shows that GCG, like EGCG, significantly suppressed the UVB-induced decrease in collagen fibers, compared to NC (Table 1), suggesting that GCG protected skin from UVB-induced photoaging via improving collagen functions.

Appropriate levels of skin moisture and skin lipid play a key role in the structure of stratum corneum and skin barrier function. Formulations containing marine sponge improved skin quality by increasing skin hydration and skin lipids^33^. Hyaluronic acid, prolyl-hydroxyproline and hydroxyprolyl-glycine maintained skin moisture, resulting in improvement of skin conditions such as increase in elasticity and decrease in wrinkles and roughness^34,35^. Ethanol extract of *Alchemilla mollis* effectively prevented UVB-induced skin water loss, resulting in alleviation of skin photoaging^36^. The present study shows that the skin oil and moisture in NC and PC were significantly increased compared to BC (Table 1). However, these two indicators in ML and HL were lower than NC though they were higher than BC (Table 1), suggesting that GCG had no effects on prevention of skin oil and moisture loss. The human sebum, which is responsible for oily skin, is reported to exert a photoprotective effect and an increase in the sebum secretion contributed to an augmentation in the skin softness and elasticity^32–34^. The oil levels in the UVB irradiated skins including those treated by sunscreen lotion and GCG were increased significantly (Table 1). These suggest the increase in skin oil level is a response of sebum to the UVB irradiation. However, the oil level in skin treated by EGCG was not significantly increased compared to BC though its skin elasticity was significantly higher than that in BC and NC (Table 1). Also, the skin elasticity in ML was significantly higher than those in NC and PC though its skin oil level was lower than the latter. These can be explained as: (1) EGCG and GCG at appropriate level partially absorbed the irradiated UVB where the sebum showed less response to the low level UVB. It is well known that catechins including EGCG and GCG have strong absorption around the wavelength of UVB. That is why the catechins are detected at 280 nm in quantitative studies. (2) The levels of collagen fibers in EGCG and ML were significantly higher than NC and LL, which might partially contribute to the skin elasticity.

In response to UVB irradiation, there was a marked increase in the number of melanosomes (Figs. 2 and 3), which was consistent with the previous study^37^*.* The suppressive effects of GCG on melanosomes formation induced by UVB (Figs. 2 and 3, Table 2) explain why GCG treatment decreased the pigmentation (Table 1). Many studies show that inhibition of tyrosinase activity contributes to the suppression of skin pigmentation. Turmeric extract, salidroside and paeonol had potential for preventing pigmentation induced by UVB radiation via inhibiting the tyrosinase activity and melanin synthesis, and they were used as melanin formation inhibitors for hyperpigmentation therapy^38,39^. Molecular docking showed that GCG and EGCG bound to the active center of tyrosinase and interacted with copper ions and key amino acid residues via hydrogen bonding and hydrophobic interactions, resulting in a looser conformation of tyrosinase and decrease in tyrosinase activity, during which the half maximal inhibitory concentration (IC_50_) was 36.8 ± 0.21 μmol/L for GCG and 39.4 ± 0.54 μmol/L for EGCG^40,41^. These suggest that GCG alleviated UVB-induced melanosome formation via suppressing tyrosinase activity.

The skin is a high turnover organ whose constant renewal depends on the rapid proliferation of its progenitor cells. The energy required for these metabolically active cells is supplied by mitochondrial respiration^42^ and so mitochondria play innate role in maintaining skin homeostasis and dysfunctions of mitochondria will lead to skin disorders. During mitochondrial respiration, reactive oxygen species (ROS) are inevitably produced, in which the ROS disrupts macromolecular and cellular structures if it is not quenched appropriately by the antioxidant system. The mitochondrial ROS-induced oxidative damage is the molecular basis for diverse pathophysiological issues including skin photoaging. Aberrations in the mitochondrial DNA (mtDNA) frequently take place in photoaged skin lesions in which skin homeostasis and pigmentation are affected. The mitochondrial dysfunction is the direct consequence of UV-induced skin photoaging. Improving mitochondrial functions will be effective against skin photoaging and some skin diseases, and so therapeutic targeting in the skin, either via ATP production boost or free radical scavenging, has gained attention from clinicians^43^. Mitochondrial membrane weakening triggers the autophagic process^44^, which removes the damaged mitochondria induced by UVB irradiation. The mitophagy is one of the important repair mechanisms for UVB-induced damage^45^. Anti-UVB agents such as nitrogen-phosphorous-doped carbon dots (NPCDs) induced the activation of autophagy by upregulating the protein expression levels of LC3-II and autophagy-related-5 (ATG-5)^46^. EGCG protected skin against UVB induced photodamages by suppressing mitochondrial dysfunction^47^. Glycyrrhizic acid significantly inhibited the UVB-induced skin photoaging via reducing ROS, NF-B, cytochrome C and caspase 3 levels^48^. Present study shows that obvious aberrations were observed in mitochondria of NC and LL in which mitochondrial crest and matrix density were reduced, and vacuolation was increased. There were no significant differences in mitochondrial structures between treatments of ML, HL, BC and PC, in which mitochondrial membrane and mitochondrial matrix were clearly observed. These suggest that GCG protected skin photoaging by alleviating mitochondria dysfunction induced by UVB irradiation.

Bioavailability of tea catechins is an important factor influencing their pharmaceutical effects^50^. EGCG uptake into and through mammalian skin is dependent on both the vehicle used and the length of topical exposure^51,52^. Pure EGCG dissolved in acetone applied topically to mouse skin prevented UVB-induced photocarcinogenesis, whereas oral dosing is inactive^51,53^. Nevertheless, meta-analysis showed that oral supplementation of green tea catechins complex was highly effective at low-intensity ultraviolet radiation-induced erythema response compared to placebo and regular supplementation of green tea catechins was associated with protection against UV-induced damage due to erythema inflammation^54–57^. The absorption of EGCG dissolved in acetone into the mouse and human skins was more rapid than that in ointment formulation^52^. However, study also showed that human skin provides a much more efficient barrier than mouse skin for preventing systemic availability from topically applied EGCG^52^. Though GCG is confirmed to show a protective effect against UVB-induced mouse skin photodamages in the present study, researching an appropriate vehicle for GCG formula is still a topic in the future.

## Conclusion

GCG protected hairless mice from UVB-induced skin photodamages by improving skin elasticity and collagen fibers, and inhibiting aberrations in mitochondria and formation of melanosomes. Because GCG is more chemically stable than its epimer counterpart EGCG^49^ and it is not susceptible to environmental light, heat and oxygen, which endow it a good ingredient for sunscreen formula.

## Supplementary Information

Supplementary Information.
