# Supplementary material for: Protective effects of gallocatechin gallate against ultraviolet B induced skin damages in hairless mice
Source: Sci Rep. 2022 Jan 25;12:1310. doi: 10.1038/s41598-022-05305-9 (PMC8789851; doi:10.1038/s41598-022-05305-9)
Supplement: Supplementary file 1 — Supplementary Information. [file 41598_2022_5305_MOESM1_ESM.docx]

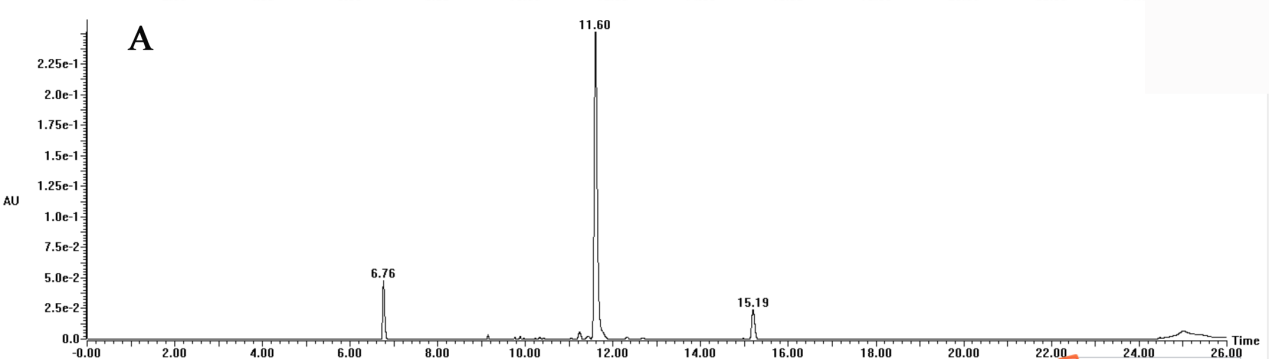


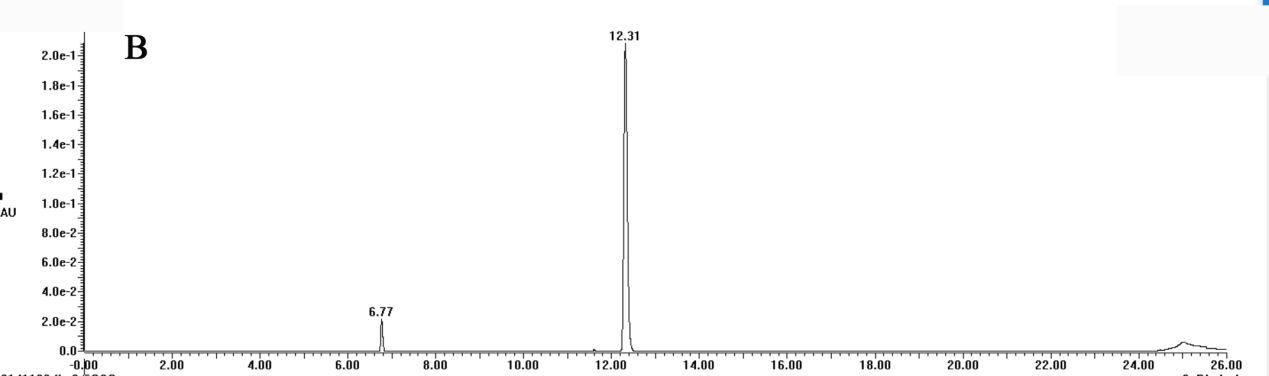


**Figure S1. HPLC profiles of EGCG and GCG used in the present study**

A: EGCG, at retention time 11.60 min; B: GCG, at retention time 12.31 min
